# Culturally-adapted cognitive behavioural therapy based intervention for maternal depression: a mixed-methods feasibility study

**DOI:** 10.1186/s12905-019-0712-7

**Published:** 2019-01-28

**Authors:** Sobia Khan, Karina Lovell, Farah Lunat, Yumna Masood, Sadia Shah, Barbara Tomenson, Nusrat Husain

**Affiliations:** 1 The University of Manchester, 3rd Floor (East), Jean McFarlane Building, University Place, Oxford Road, Manchester, M13 9PL UK; 2 Division of Nursing, Midwifery and Social Work, The University of Manchester, Room 6.322a, Jean McFarlane Building, University Place, Oxford Road, Manchester, M13 9PL UK; 3 Lancashire Care NHS Foundation Trust, The Mount, Whalley Road, Accrington, BB5 5AD UK; 4 Cumbria Partnership Foundation Trust, Garburn House, Westmoreland General Hospital, Burton Road, Kendal, LA97RG UK; 5 Centre for Biostatistics, School of Health Sciences, The University of Manchester, Manchester Academic Health Science Centre, Manchester, M13 9PL UK; 6 Lancashire Care NHS Foundation Trust, The University of Manchester, 3rd Floor (East), Jean McFarlane Building, University Place, Oxford Road, Manchester, M13 9PL UK

**Keywords:** Maternal depression, Cognitive Behavioural therapy, Cultural adaptation, British Pakistani women, Group psychological intervention

## Abstract

**Background:**

British Pakistanis are one of the largest ethnic minority groups living in the UK, with high rates of maternal depression being reported in this population. Evidence suggests that culturally-adapted Cognitive Behavioural Therapy (CBT)-based interventions for depression, may improve clinical outcomes and patient satisfaction. This study was conducted to develop and test the feasibility and acceptability of a culturally-adapted, CBT-based, manual-assisted intervention in British Pakistani mothers experiencing maternal depression.

**Methods:**

A mixed-method feasibility study that included qualitative interviews followed by the development of a CBT-based intervention for mothers with mild to moderate depression. Following the qualitative interviews, a CBT-based intervention called the Positive Health Program (PHP) was developed and delivered consisting of 12-weekly sessions. A before and after design was used to explore the feasibility and acceptability of the Positive Health Programme.

**Results:**

A culturally-adapted CBT-based group intervention (PHP) was acceptable to this group and improvements were reported in depression and health-related quality of life. The women’s understanding of ‘depression’ as a general consensus was in physical terms, but with an onset triggered by psychosocial causes. The most commonly reported factors contributing to depression were marital disharmony, lack of social support, and financial difficulties. Past help offered was primarily antidepressants, which were not welcomed by most of the women. A lack of availability of culturally sensitive interventions and the limited cultural sensitivity of NHS staff was also reported.

**Conclusion:**

This study provides preliminary evidence for the feasibility and acceptability of a CBT-based culturally-adapted group psychological intervention for British Pakistani mothers.

**Trial registration:**

Study ethics registration number: 10/H1005/62 (University of Manchester).

## Background

Maternal depression affects women in all nations and across cultures [1–4]. In the ‘Saving brains’ project, Grand Challenges Canada have emphasised on better maternal health, better protection and responsive caregiving for improving early child development, particularly in the first 1000 days of life of the child [5]. Research has identified a lack of social support and marked difficulties particularly in marital and close relationships as key factors for maternal depression [6, 7]. This is reported to be particularly evident in Pakistani [8] and British Pakistani women [6, 7]. The consequences of maternal depression are significant; it may inhibit a mother’s ability to respond sensitively to infant cues [9, 10] which is vital for infant growth and development [11], and may result in poor cognitive [12, 13] psychosocial and physiological outcomes [14, 15]. In addition, research in middle and low income countries has suggested an association between maternal depression and child growth impairment [16–18].

Explanatory models of illness encompass a person’s ideas about the nature of their problem, its cause, severity, and prognosis and treatment preferences [19]. Disagreement between patients’ and professionals’ explanatory models may affect help-seeking behaviour and treatment adherence. A study conducted by Karasz reported that South Asian immigrant women viewed depression quite differently from White women [20]. White women attributed depressive symptoms to a biological cause such as “hormonal imbalance” or a “neurological problem” whereas South Asian women interpreted the symptoms as a reaction to “life problems” or “situational stress” [19].

An experimental vignette study conducted by Burt and colleagues identified that British Pakistanis have higher General Physician (GP) consultation rates compared to other ethnic groups [21]. Despite this evidence, a majority of South Asian mothers with common mental disorders are not identified by their GPs [22]. This may be due to difficulties in communication, cultural differences in presentation of symptoms [22], or different explanatory models between the physician and the patient, which often results in the patient remaining undiagnosed and untreated [23, 24]. Another possibility is cultural stereotypes that can mislead diagnosis and different treatment pathways [25].

Gater and colleagues reported the efficacy and acceptability of a culturally-adapted social group intervention [26] in British Pakistani women. It was reported that the social functioning and satisfaction levels of the women improved significantly with social treatment as compared to medical treatment only [26]. Evidence suggests that psychological interventions such as Interpersonal Psychotherapy (IPT) and CBT are effective in the treatment of maternal depression. [27, 28]. In order to improve the diagnosis and treatment of maternal depression, it is important to understand the experience of depression in British South Asian women. This study presents British Pakistani women’s experiences of depression, their explanatory models about depression, and their views about the type of help they may find acceptable, through in-depth interviews. The study also explores the acceptability and feasibility of a CBT-based, manual-assisted intervention called the Positive Health Program (PHP) in this group of women.

## Methods

### Study design

This was a mixed-methods feasibility study. Initially qualitative interviews were conducted to explore the women’s experiences of depression, their explanatory models about depression, and most appropriate help they may find acceptable. These women were invited to take part in the feasibility study, with assessments before and after the group intervention to compare the differences in severity of depressive symptoms and quality of life scores. Mothers were approached and screened using the Edinburgh Postnatal Depression Scale (EPDS) [29] after informed consent was obtained.

#### Inclusion criteria

British Pakistani mothers aged 18 years and above, scoring ≥12 on EPDS.

#### Exclusion criteria

Women diagnosed with physical or learning disability, postnatal or other psychosis or actively suicidal.

### Sample

Those British Pakistani mothers, who had taken part in our earlier cohort study on social stress and depression in the ante-natal and post-natal period [6], were approached for participation in this study. Out of the total 47 depressed mothers identified residing in Central Manchester, only 36 could be contacted, so they were approached by the research assistant and given information about the current study. The recruitment of participants took place from October 2008 to February 2009. Participants were assessed by a trained bilingual researcher fluent in English and Urdu. The assessments took place at the participants’ homes. Following informed consent, the 36 women were screened for depression using the Edinburgh Postnatal Depression Scale (EPDS) [29]. Out of the 36 participants, 18 women scored ≥12 on the EPDS, with the diagnosis of depression being confirmed using the Clinical Interview Schedule – Revised (CISR) [30]. Out of the eighteen participants, three opted out due to work commitments and time constraints and a total of 15 women agreed to take part in the study.

### Data collection and analysis of the qualitative interviews

Initial data was collected through in-depth interviews. The interviews topic guide was developed through discussion within the research team and the existing literature. Key areas explored were, perceived causes of maternal depression, what help women had previously received for maternal depression and the type of help they would like to receive. All interviews were digitally recorded and transcribed verbatim. Data were analysed using framework analysis, consisting of familiarisation of the data, identification of a theoretical framework, indexing, charting, mapping and interpretation. Following detailed readings of the transcripts, themes and sub-themes were identified and a thematic framework was developed. The coding framework was then applied manually to the interview transcripts and then pasted to the excel spread sheets. Using the spread sheets, the study team (SK & KL) compared and contrasted various subthemes to finalise the theoretical table.

### Development of the intervention

The development of the group psychological intervention, involved a process by which findings from multiple data sources and methods were combined to obtain in-depth understanding of the specific issues in designing and delivering the proposed culturally-adapted intervention for British Pakistani mothers. Data was reviewed and synthesised, with this process relying heavily on the information obtained in the earlier conducted qualitative interviews which explored the views about a feasible and culturally acceptable psychological intervention for depressed British mothers of Pakistani origin. The group sessions were conducted at a Children’s Sure Start Centre in Central Manchester to provide an easily accessible location with appropriate childcare facilities for mothers.

### The intervention (Positive Health Programme)

The culturally-adapted group intervention is based on the principles of Cognitive Behavioural Therapy (CBT). The study participants decided to call it the Positive Health Programme (PHP) (Table 1). The programme involved 12 weekly sessions facilitated by a research therapist who led the group with some training in CBT and leading depression groups using CBT skills and principles. Regular supervision was provided by a senior CBT therapist (KL) and a senior psychiatrist (NH). The sessions lasted approximately 60–90 min and involved group discussions, case scenarios, individual goal setting, and skill-based activities. The programme was designed to be simple and pragmatic which could be delivered by an individual trained in mental health and the principles of CBT [31]. The focus was on keeping the programme culturally appropriate. The key elements covered in the manual are detailed in Table 1 (the manual is available on request).

**Table 1:** Elements of the Positive Health Programme (PHP)

Positive Health Programme (PHP)		
Theoretical basis	Based on principles of cognitive behavioural therapy (CBT)	Based on the previously mentioned findings and the literature review of empirical culturally based therapies for depression, cognitive behavioural therapy (CBT) was taken as the most suited approach that could be adapted for use with British Pakistani women. The ‘here and now’ problem solving CBT approach was felt to meet the requirements reported above and in the qualitative interviews.
Delivery	Minimally trained clinical staff, health research facilitators, mental health graduates such as assistant psychologists, IAPT psychological wellbeing practitioners. Intervention is simple enough to be delivered by anyone trained in mental health.	Rahman (2007) devised the following requirements for delivery: 1. Participant = family level • Should focus on overall maternal health rather than maternal depression • Should focus on the identified maintaining factors for depression • Should be active and empowering • Should be skill based 2. Delivery and facilitation level • Should be culturally sensitive • Should be simple and pragmatic • Should avoid stigmatisation 3. Health system level • Should be evidence-based • Should move away from ‘medical model’ to a ‘Psychosociospiritual’ model • Should be community based • Should be culturally adapted • Should be cost effective
Structure of the intervention	12 sessions, each session approximately 60–90 min	Group CBT based manual with step-by-step instructions for conducting each session. Activity workbooks and hand-outs for mothers
Structure of the group	Group welcoming and connecting, introduction to the session topic, group discussion, engaging in skill based activities, planning individual goals, and homework setting session	On-going training and supervision by clinical staff including a senior CBT therapist and a senior psychiatrist
Areas covered in the sessions/manual	• Identifying the pressures and expectations of being a British Pakistani woman • Understanding and managing self-esteem • “Keeping up with the Joneses” “Chaudhry’s” • Exercise, looking good and ways of building motivation • Religion and spirituality • Relaxation: “Taking time out” • Culturally sensitive assertiveness and confidence building • Breaking Social isolation and building social networks • Practising CBT and assessing change	The ABC model of CBT was used to demonstrate the relationship between thoughts, feelings and behaviour in each session. Specific interventions included psycho-education, behavioural activation, problem solving, relaxation, and managing negative thoughts.

### Assessments

Participants were assessed at baseline, 3 months (end of intervention), and 6 months after baseline. Uptake, adherence, and acceptability were chosen as the feasibility parameters for this study. These were measured by the participants’ attendance records for the group sessions. Participants who were absent were followed-up to explore reasons for their absence.

### Edinburgh postnatal depression scale (EPDS) [18]

The presence of depression was assessed with EPDS at baseline, week 4 and week 8 of the intervention, end of intervention (3 months) and 6 months after the baseline assessments. EPDS is a 10-item, self-report scale and has been used among women in Pakistan [32] and British Pakistani women [31]. The EPDS has been validated in this population and results suggest that the EPDS has good psychometric properties [33].It has also been used and validated with mothers 0 to 3 years of children [34, 35].

### The clinical interview schedule revised (CIS-R) [30]

This structured interview was used to confirm the diagnosis of depression. It has 14 sub-sections and has been previously used with British Pakistani women [26].

### EuroQol (EQ-5D) [36]

The EQ-5D provides a descriptive profile of health-related quality of life and a subjective overall rating of the participant’s own health status on the day of administration by means of a visual analogue scale. In an earlier UK based study, the EQ-5D was used with British South Asians in a primary care setting [37].

### The dyadic adjustment scale (DAS) [38]

This 32 item self-report measure was used to assess marital relationship. Four factors are reported: dyadic satisfaction, dyadic consensus, dyadic cohesion, and affection expression. This scale has not been used previously with British Pakistani depressed mothers.

### Multidimensional scale of perceived social support (MSPSS) [39]

This is a 12-item instrument designed to assess perceptions about support from family, friends and a significant other. The scores for each item can range from 1 to 7. A high score indicates high levels of perceived support. In a study of maternal depression in Pakistani women, MSPSS was found to have good construct validity, and the internal consistency (Cronbach’s alpha) of MSPSS was 0.92 [40].

### Satisfaction

This 6-item adapted scale called “Satisfaction with support and treatment” is extracted from the full-scale Verona Service Satisfaction Scale [41], which was used in the previous depression trial with British women of Pakistani origin [26]. At the end of the intervention, participants were asked for their self-rated satisfaction with treatment based on a three-point ordinal adapted scale derived from the Verona Service Satisfaction Scale [41].

### Statistical methods

Since the sample size was too small to confirm the data distribution, non-parametric tests were used for all measures. Median scores at all time-points for each score are presented, and changes from baseline to all follow-ups have been assessed using the Wilcoxon matched pairs signed-ranks test.

### Ethical approval

Ethical approval was granted by the Local Research Ethics Committee and the University of Manchester (registration number 10/H1005/62).

### Cultural adaptations

Previous research by our group and our colleagues has shown that adaptations need to be implemented within CBT for it to be effective for South Asian women. A study by Naeem et al. [42] reported that these adaptations need to focus on three main areas, i) awareness of the cultural issues and getting ready for relevant therapy ii) assessments and engagement of the population with the therapy iii) adjustment in therapy. Therefore, adaptations in this study were done in-line with the above mentioned study [42], with some guidance from other studies by our colleagues, such as the study on cultural adaptation of CBT for psychosis for British South Asians [43], guidance from culturally sensitive CBT for depression in Pakistan [44] and also our other work in Pakistan [33, 35]. With the help of qualitative interviews and understanding explanatory models about depression and suggestions given by mothers about the type of acceptable help the central adaptations made to the manualised intervention were; South Asian CBT jargon, culturally appropriate assignments and homework, folk stories and examples with considerations to religious beliefs and understanding the ABC model using culturally appropriate stories.

## Results

### Qualitative results

Fifteen of the 18 mothers who fulfilled the inclusion criteria agreed to take part in the in-depth interviews. They were interviewed at their homes; each interview lasted approximately 40–80 min. Their age ranged from 23 to 41 years with a mean age of 33 years. The women had an average of three children, ranging from 2 to 5 years. Eight women were first generation migrants and seven were born in the UK. Twelve women were living with their husbands, two were divorced and one separated. At the time of the interview, 11 women were homemakers, two worked fulltime and two worked part time.

The analysis of the data revealed three themes described below:

- The perceived causes of maternal depression
- Previous help sought for maternal depression
- The type of help needed for maternal depression

### The perceived causes of maternal depression

All women described the term ‘depression’ through a description of somatic symptoms and a psychosocial model with spiritual explanations. The main social causes reported for their depression were lack of support and marital problems, social isolation, financial hardship, bereavement, low self-esteem, and difficult family relationships particularly with their husband.

*“It’s the panic attacks, the shivers. Sometimes I’d be watching the TV and I would feel my heart thumping, and I would think hang on I’m not thinking about anything, then what’s the reason? I can unnecessarily get the panic attacks, constant migraines, three times a week. That is not normal. I don’t know what triggers it because sometimes I’m not thinking anything at all” (P1).*

#### Lack of support

Lack of social support was the most commonly reported factor for depression. A change in these women’s family structure, from living in an extended to a nuclear system, may have contributed to depression as all of the participating women in this study lived in a nuclear household, and potential support networks may have been lost.

*“I’m not getting any support or the support that I need. People do come to me, help me out but I haven’t got the support that I need. My mum just recently passed away in June. I’ve been really depressed since then. I’ve had no support”. (P10*)

#### Difficult relationship with the family

Some of the women spoke openly about the negative influences of their mothers on their mental health. Difficult relationships with not only mother in laws but also their own mothers and the mother’s lack of understanding and denial of participant’s depression often led to many of these women suffering in silence.

*“I can only talk about my mum. If she sees me crying, she says, "Why are you crying? There’s nothing wrong with you. You're just being yourself, you do this all the time, you cry for no reason, you're happy, you're here, you have a nice husband, and you have no major responsibilities like I did so pull yourself together”. (P14)*

#### Marital difficulties

Marital difficulties have been highlighted as a prominent issue in this group. Poor marital relations not only equated to a lack of practical and emotional support from the husband but often involved domestic violence. The women expressed the need to stay in a poor marriage for the sake of their children’s future and a lack of options to change their circumstances because of financial dependence on their husband.

“*When I see my life, I feel like throwing him out of the house before dark. But when I see him with the kids, I can’t do that. He is there for them. Tomorrow if I separate and I can’t provide for my kids and they go astray, what will I do? If they start staying out till late, how will I discipline them, what would I do if they don’t listen to me?” (P13)*

*"I think it’s mainly these marriages. Because in our marriages everything stays within the four walls other people don’t know what’s going on. Even though at times when we have been screaming, the voices don’t go outside". (P13)*

#### Low self-esteem

The role of low self-esteem in the development of depression was an important factor. The factors associated with low self-esteem varied from dissatisfaction with appearance to a lack of career achievements. Low self-esteem due to poor marital relationship was also evident.

*“He makes me feel bad about myself? Why am I so ugly that he doesn’t want to know me, but it’s been so many years now since we’ve been married. I was a confident person at that time, but over the years I lost it. Then I did a self-esteem course and that picked up my confidence but then I lost it again”. (P10)*

#### Religion and spirituality

Some spiritual elements were perceived to contribute to depression. Some women in this study stopped their religious practices as a result of feeling guilty and thoughts of being punished by God for their past sins. God was no longer seen as the source of support and women ended up with continuing feelings of remorse and persecution. These feelings were reported by the women as a perpetuating factor in the persistence of depression. The underlying mechanisms for the fear of punishment from God often arose from feelings of guilt for various reasons, for example, undergoing an abortion, rebelling against parent’s wishes, and neglecting elderly parents. The fear of punishment and thoughts of eternal suffering contributed to symptoms such as hopelessness and helplessness.

*“Sometimes, I think I am being punished by God for what I did to my dad, making him upset you know, getting married without his will. That’s why, even though I’ve got kids now, healthy kids, I’m still not happy”. (P11)*

#### Financial problems

Women described feeling low in mood and stressed due to financial difficulties. Their lack of financial contribution to the household caused these women to feel unworthy and disempowered, and this sometimes contributed to marital disharmony.

*“Our financial worries make me sad. We are so dependent on my husband’s brother. He supports us so much. I sometimes wish that we didn’t have to ask anyone or live on someone else’s money. It’s shameful in our community isn’t it? So I do feel very sad and ashamed in front of my other relatives. I didn’t ever imagine a life like this”. (P2)*

### Previous help sought for depression

The majority of the women were reluctant to take antidepressant medication due to side effects. They perceived anti-depressants as “numbing the pain” rather than addressing the issue. Antidepressant medication was prescribed initially to 11 of the 15 participants. However, seven out of the 11 participants were also offered counselling at some point. Four out of the 15 participants had never approached the GP for any type of help for their depression as it was seen futile and the type of help that was available was not perceived as useful.

Out of the seven participants who were offered counselling, only three attended the counselling sessions. The counselling was seen as helpful to a certain extent; however, it lacked cultural knowledge and sensitivity which in turn made all three of the participants not attend further sessions. These women emphasised the importance of having a culturally sensitive therapist belonging to the same culture as they were.

“*I got treatment at that time, when I had my first child. I was really bad at that time. I was depressed all the time, just really bad. It got worse with my second child. They gave me some antidepressants. I didn’t really take a lot of them. I didn’t want to. I just didn’t want to take any tablets.” (P11)*

### The types of help British Pakistani women want for maternal depression

The majority of the women favoured a group psychosocial treatment to reduce social isolation and to increase social support. Although women wanted the treatment to have a supportive element, support alone was not seen as sufficient to overcome depression. The women expressed the need for a more directive and problem focussed approach and wanted help to improve self-esteem and self-confidence, to learn coping skills such as assertiveness to improve marital relationships, problem solving, time management, relaxation, and anger management. The participants also wanted help with managing negative thoughts.

*“I would love that (group treatment), just to share your emotions with people, talk to people because I can’t just go out there and talk to anybody. I can’t find anyone who can mix with me” (P11).*

Most women showed an interest in exercise, building social networks, practising religious activities and emphasised that religious activities should be incorporated in modern day psychosocial interventions. The women believed that prior to the onset of depression their faith in religion had helped them to cope better with life difficulties.

The lack of appropriate support with marital difficulties was raised by women who were experiencing marital problems. These women highlighted the need for some specific services for Pakistani men as they were seen as the main cause of their depression. According to these women, not enough is being done to engage Pakistani men into treatments which may help with marital problems.

The perceived barriers to attending treatment were lack of transportation, unavailability of childcare, language, workload, time of day, and often the husband. Because of lack of autonomy to movement and decision making over half of the women believed that their husband would prevent them from receiving any treatment for depression. There is lot of stigma about mental health and concerns about the community grapevine. This largely relates to the concept of ‘Izzat’ meaning family honour that is very important in Pakistani families. Keeping face and maintaining the family honour results in a lot of things being kept hidden and a need to maintain appearances within the community and thus issues such as mental health problems can be covered up.

It was suggested that the treatment for depression should be advertised to wider family members as an educational course for positive ways of coping with motherhood and stressing the beneficial outcomes for children. This was important because if the focus is on the child rather than the mother it would be more acceptable to the extended family and the wider community and; thus, there will be little stigma attached which could affect the “honour” of the family. It was also suggested that promoting interventions in such a way may also help to reduce the stigma associated with mental health issues and treatment for mental health problems [29]. Therefore, a non-stigmatising name for the therapy was considered important.

*“I mean if my husband finds out that this is a group for (depressed) women, he’s not going to let me come to it.” (P11).*

### Quantitative results

#### Demographics

Fifteen women consented to participate in the intervention, out of those 10 attended 4 or more group intervention sessions. They were aged between 25 and 40 years (mean age, 33 years), 8 were married, one was divorced and one was separated. The mean number of children was 3, ranging from 2 to 5, all except one was a homemaker, 5 were first-generation migrants and five were second-generation migrants. All participants were educated up to at least GCSE.

#### Attendance at the positive health Programme (PHP)

Attendance levels were used to assess the acceptability of the intervention. The PHP group intervention was designed to have 12 sessions, but one session was cancelled due to unavailability of a room at the children’s centre. The content of this session was incorporated in the remaining sessions. A total of 10 of the 15 participants attended 4 or more sessions. Two attended less than 4 sessions and 3 did not attend any of the sessions. The group attendance was noted by one of the group facilitators, the absentees were followed and a note of reasons for their absence was made. Of the three women who did not attend any session, one was physically ill, second was back in full-time work and third could not make travel arrangements.

After developing consensus with our colleagues working on culturally adapted CBT and brief psychological interventions [44–46] it was decided that those women completing four or more sessions would be termed as ‘completers’.

#### Depressive symptoms

Reduction in depressive symptoms was measured using the EPDS at 5 different time-points: baseline, intervention week 4 and 8, end of intervention and 6 months after baseline assessment. The median EPDS score at baseline was 20 which reduced to 11.5 by week 4 and to 11 by week 8. The drop in EPDS scores was sustained at 6 months after baseline with a median score of 5.

#### Service satisfaction

Of the total participants (*n* = 15), 10 attended 4 or more sessions and were called ‘completers’. The majority of completers were satisfied with the intervention; four highly satisfied, five fairly satisfied and one not satisfied. Six of them said they would ‘definitely recommend’ this therapy to others and four said they ‘might recommend’.

#### Marital relationships

The DAS scores showed significant improvement (*p* = 0.01) in marital relationships with the intervention. The mean DAS score at baseline was 55 (SD = 30.3), which increased to 78 (SD = 34.8) at the end of intervention, and was sustained at 76 (SD = 32.1) at 3 months post-intervention follow-up.

#### Social support

There was an increase (indicating improvement) in total MSPSS scores in each of the three subscales from baseline to end of treatment, which was maintained at 6-month follow-up (Table 2). This increase in the scores reflected increase in perceived support from family, friends, and significant others.

**Table 2:** Median MSPSS Scores across Three Time-Points

Subscale	Baseline median score	EOI^a^ Median Score	*p*-value for improvement from baseline to EOI	6-Month Median Score	*p*-value for improvement from baseline to 6-month follow-up
Family	15	20.5	0.005	20	0.016
Friends	13	21	0.009	20.5	0.005
Significant other	11.5	22	0.008	19.5	0.012
Total score	40.5	60.5	0.005	59.5	0.005

^a^ *EOI- End of intervention - (3 months)*

#### Quality of life scores

EQ-5D score showed a significant improvement, in health status of mothers, with the intervention. EQ-5D scores improved from baseline (median = 55) to end of treatment (median = 72.5, *p* = 0.036), and was maintained at 6-month follow-up (median = 70, *p* = 0.037).

## Discussion

Feedback from the brief, adapted Verona Service Satisfaction questionnaire showed that the Positive Health Program (PHP) is an acceptable and feasible intervention for British Pakistani mothers. Two-third of the women (10/15) completed the intervention and attended four or more sessions. Maternal depression has been reported as hard to treat because of its longstanding and complex nature [47]. Considering this is a hard to engage population, the attrition rate for this group intervention was relatively low (66% were completers), similar to that reported in a previous study with persistently depressed British Pakistani mothers in which 50% of the mothers were completers [48]. Several factors such as using familiar language, culturally aware therapists and facilitators, childcare facilities, and transport assistance helped to engage and retain participants in the study. All these factors were initially identified through qualitative interviews.

A key factor in engaging and retaining participants was engagement with their families. In a previous study, British Pakistani women [26] reported that a major barrier to social group participation was resistance from family members, particularly husbands. Hence; this intervention was presented to the families from the perspective of improving not only the health of the mother, but possibly also producing beneficial outcomes for the child. The acceptability of the intervention may have been enhanced by the availability of transport and free childcare. Earlier studies showed lack of transport to be a reason for drop outs [26, 49, 50]. Similarly, free crèche service was provided in a room adjacent to the PHP group intervention room, mothers were free to go and check their children as they pleased. The intervention was conducted during the school term to attract mothers with school-going children and to minimise dropouts. Reay and colleagues’ pilot intervention study of a group interpersonal psychotherapy for postnatal depression reported similar findings [51].

A positive relationship with the group facilitators was found to be a vital factor for participation in the study. Chaudhry and colleagues also reported the importance of a healthy relationship between facilitator and mothers for successful intervention delivery [48]. Another key feature of this study is the cultural acceptability of the intervention and the facilitators’ same ethnic background, which is reported to be important in previous studies as well [26, 52].

Women in this study did not receive the type of help they aspired for from the National Health Service (NHS). It is interesting to note that although the women described depression in physical terms, they provided no medical explanations to explain their condition rather all their explanations were psychosocial in nature. One of the research programs in Goa, India, looking at maternal health reported that although women described a number of somatic complaints, there was no ‘denial’ of their social and emotional contexts [53]. Regarding help for depression the findings suggest the need to develop culturally appropriate interventions and possibly starting with discussing somatic symptoms to cater for the needs of depressed British Pakistani women. At the end of the intervention, the participants in this study reported improvement in symptoms of depression. These findings are in line with Chaudhry et al. [48] who reported women in their study eagerly looked forward to attending the groups and used the expressions “mood became fresh” and “forgot our problems” to describe their positive experience which also resulted in enhanced self-confidence.

Our study suggests that depression is perceived as a reaction to unfavourable life events with a psychosocial aetiology rather than a biomedical model. The perceived causes of depression are similar to previous studies in the UK and those reported from Pakistan [22, 54]. A study from the UK reported that Black women were more likely to seek help from spiritual sources for depression than white women and reported spirituality as a coping mechanism [55]. There is evidence from earlier studies that women who lacked social, emotional and/or practical support continued to experience depression [31, 56].

Similar to the finding of Reay and colleagues [51], this study also showed improvement in marital relationships which might be attributed to exercising greater control over emotions and developing a positive attitude. The majority of women attributed marital problems as a significant factor for their depression. It has been suggested that marital difficulties can even lead to self-harm and suicide in South Asian women [57, 58]. These results warrant attention as some studies suggest that South Asian women have higher rates of suicide compared to White women living in the UK [54, 58], particularly those with an affective disorder [59].

We found British Pakistani mothers’ conceptualisation of depression to be very useful in guiding the development of group psychological treatment for depression. A majority of the women in the study favoured group intervention to address social isolation some suggested if individual therapy was also made available to offer flexibility and to maximise participation. Counselling services were found to be helpful in terms of having, “Someone to talk to”, but women favoured problem solving approaches for the treatment of depression. These women described a degree of disempowerment and asked about ways of improving their self-esteem and self-confidence. However, there are certain cultural issues we need to be aware of, though these women described the need to become more empowered; they wanted this within the domains of the Pakistani culture. The study suggests a need for culturally sensitive treatment as opposed to standard ‘usual care’. The women described health professionals as “lacking in knowledge and understanding of the Pakistani culture” and not understanding the underlying root causes. In a healthcare setting this would imply that staff should be trained in cultural awareness so that the women are able to say what they are thinking without feeling they may not be understood. Recent reports indicate inequalities in maternal health and the need for tailored maternity services to improve access to care for women from ethnic minorities [60].

### Limitations and future research

The major limitation of the study is small sample and the non-controlled pre-post feasibility study design. Another limitation of the study is the difficulty encountered with some of the outcome assessment tools. When using the Dyadic Adjustment Scale tool, the women were reluctant to share certain personal information about the nature of their relationships with their husbands. Most women in this study believed that the intimate nature of a marital relationship should not be discussed outside of the marital relationship. We didn’t collect data on the number of mums taking the antidepressants at any stage during the intervention or by the end of the intervention. Also, we were unable to determine if the mothers had persistent PND depression or if it was relapse of PND.

In addition, the study took place in one geographical area in England; hence, these results may not be generalisable to other regions and populations. The participants were from a Pakistani background, so the results may not be applicable to the other ethnic groups. A further limitation is selection bias as the sample was selected from an earlier existing cohort of British Pakistani women who may be more motivated to participate in research compared to other women attending primary care centres. In addition to the issues mentioned above, there is a need for future trials to measure self-esteem. This emerged as an important aspect during the delivery of the intervention.

Despite the fact that it was a hard to reach population to work with, two-thirds of the sample completed the intervention (10/15) and remained in the study till the end of the intervention. This also provides an estimate in terms of recruitment for future trials that a third of the participants may drop out of such interventions.

## Conclusions

This study provides preliminary evidence for the feasibility and acceptability of a CBT-based culturally-adapted group psychological intervention for British Pakistani mothers. The participants found the culturally relevant psychological intervention acceptable and felt that the group sessions provided social support and helped to gain self-confidence. The participants reported that a more directive and problem focussed approach helped them to develop skills such as assertiveness, relaxation, and anger management. Reduction in depression and improvement in marital relationships, social support and overall health status mothers were noted but findings are limited due to the small sample size and no control group.

## Abbreviations

CA-CBT: Culturally Adapted Cognitive Behaviour Therapy
CBT: Cognitive Behaviour Therapy
CISR: Clinical Interview Schedule – Revised
DAS: The Dyadic Adjustment Scale
EPDS: Edinburgh Postnatal Depression Scale
GP: General Physician
IPT: Interpersonal Psychotherapy
MSPSS: Multidimensional Scale of Perceived Social Support
PHP: Positive Health Programme
PND: Post-natal Depression
TAU: Treatment as Usual
